# Population dynamics and drug resistance mutations in *Plasmodium falciparum* on the Bijagós Archipelago, Guinea-Bissau

**DOI:** 10.1038/s41598-023-33176-1

**Published:** 2023-04-18

**Authors:** Sophie Moss, Emilia Mańko, Hristina Vasileva, Eunice Teixeira Da Silva, Adriana Goncalves, Ashley Osborne, Jody Phelan, Amabelia Rodrigues, Paulo Djata, Umberto D’Alessandro, David Mabey, Sanjeev Krishna, Anna Last, Taane G. Clark, Susana Campino

**Affiliations:** 1 Faculty of Infectious and Tropical Diseases, London School of Hygiene and Tropical Medicine, London, UK; 2 Ministry of Public Health, Bissau, Guinea-Bissau; 3 Bandim Health Project, Bissau, Guinea-Bissau; 4 National Malaria Control Programme, Ministry of Public Health, Bissau, Guinea-Bissau; 5 MRC the Gambia Unit, Fajara, The Gambia; 6 Clinical Academic Group, Institute for Infection and Immunity, and St. George’s University Hospitals NHS Foundation Trust, St. George’s University of London, London, UK; 7 Centre de Recherches Médicales de Lambaréné (CERMEL), Lambaréné, Gabon; 8 Institut Für Tropenmedizin Universitätsklinikum Tübingen, Tübingen, Germany; 9 Faculty of Epidemiology and Population Health, London School of Hygiene and Tropical Medicine, London, UK

**Keywords:** Malaria, Genomics

## Abstract

Following integrated malaria control interventions, malaria burden on the Bijagós Archipelago has significantly decreased. Understanding the genomic diversity of circulating *Plasmodium falciparum* malaria parasites can assist infection control, through identifying drug resistance mutations and characterising the complexity of population structure. This study presents the first whole genome sequence data for *P. falciparum* isolates from the Bijagós Archipelago. Amplified DNA from *P. falciparum* isolates sourced from dried blood spot samples of 15 asymptomatic malaria cases were sequenced. Using 1.3 million SNPs characterised across 795 African *P. falciparum* isolates, population structure analyses revealed that isolates from the archipelago cluster with samples from mainland West Africa and appear closely related to mainland populations; without forming a separate phylogenetic cluster. This study characterises SNPs associated with antimalarial drug resistance on the archipelago. We observed fixation of the PfDHFR mutations N51I and S108N, associated with resistance to sulphadoxine-pyrimethamine, and the continued presence of PfCRT K76T, associated with chloroquine resistance. These data have relevance for infection control and drug resistance surveillance; particularly considering expected increases in antimalarial drug use following updated WHO recommendations, and the recent implementation of seasonal malaria chemoprevention and mass drug administration in the region.

## Introduction

Despite knowing the causes of malaria for over a century, this disease is still a major public health problem. The World Health Organization (WHO) Global Technical Strategy aims to reduce the global malaria burden by 90% by 2030^1^. Considerable progress has been made. However, the decline in malaria mortality has slowed since 2014, and malaria deaths increased to an estimated 627,000 in 2020. This was a 12% rise from 2019, with most deaths caused by the parasite *Plasmodium falciparum*^1^. To meet the WHO target by 2030, novel strategies and tools for malaria elimination are needed.

Recently updated WHO recommendations suggest that antimalarials will be increasingly deployed for the control and elimination of *P. falciparum,* on a mass scale. These recommendations included expanding the use of intermittent preventive treatment in pregnancy with sulphadoxine-pyrimethamine (IPTp-SP) to all pregnant women, broader use of perennial malaria chemoprevention (PMC) and seasonal malaria chemoprevention (SMC), and expanded guidance for the use of mass drug administration (MDA)^2^. As a result of these recommendations, the mass use of antimalarials is likely to increase in a variety of transmission settings at the discretion of relevant control programmes. Molecular surveillance is a useful tool for evaluating the impact of mass antimalarial drug use through monitoring malaria prevalence, parasite population dynamics, and genomic markers of drug resistance.

Endemic island communities are a unique landscape within which to test malaria interventions, including MDA, due to their isolated geography. Understanding the population dynamics and genetic variation of *P. falciparum* populations on islands provides crucial information for the evaluation of these interventions, as well as routine malaria surveillance. Improved whole genome sequencing technology, including the development of Next Generation Sequencing (NGS), has dramatically reduced the time and cost of genomic sequencing through enabling the sequencing of millions of reads in parallel^3^. The subsequent increase in the generation and availability of genome sequence data allows for high resolution analysis of the genomic architecture of *P. falciparum* populations.

The Bijagós Archipelago is a group of 88 islands and islets situated off the coast of Guinea-Bissau, West Africa. The islands are isolated from mainland Guinea-Bissau; separated by 70 km of sea and accessible by ferry, which takes four hours and sails once each week. Currently, malaria interventions in the Bijagós include enhanced case finding, intermittent preventive treatment in pregnancy (IPTp) and long-lasting insecticidal nets (LLINs)^4^. Despite good coverage and adherence to these interventions, including maximal control efforts using LLINs, malaria case incidence and mortality both increased by between 5 and 25%, from 2015 to 2020^1^. Although *P. falciparum* populations have been studied in mainland Guinea-Bissau, there has been no previous research conducted on the genomic architecture of *P. falciparum* parasites on the Bijagós Archipelago. This is vital for researchers to be able to understand the impact of malaria interventions within the region. Eighteen of the islands within the archipelago are inhabited year-round, with a total population of around 25,000 people^5^. The majority of the population live in forest villages and are largely hunter gatherers with some subsistence farming and fishing^4^. The islands have a rainy season between June and November and a dry season from December to May, with peak malaria transmission occurring at the end of the rainy season in October and November^6^. Malaria prevalence on the Bijagós is seasonal and stable, with low to moderate transmission intensity. Most malaria cases are asymptomatic. A malaria prevalence survey was conducted on the most populated island within the archipelago in August 2017, during the start of the malaria transmission season. This was a cross-sectional survey of the population, which identified the prevalence of *P. falciparum* infection to be 16.9% by qPCR^5^. Persistent circulation of subclinical parasitaemia makes reducing malaria transmission very difficult in this setting. This is because asymptomatic malaria cases are a major driver of malaria transmission, and asymptomatic individuals do not generally seek treatment^5^.

One of the main challenges of whole genome sequencing (WGS) clinical malaria parasite isolates from dried blood spots is obtaining sufficient high-quality parasite DNA from infected individuals. This is particularly challenging when there is low parasitaemia in asymptomatic infections, and when working with dried blood spot (DBS) clinical samples, as the majority of DNA extracted from DBS is human. To address this, selective whole genome amplification (SWGA) can be used to selectively amplify *P. falciparum* DNA directly from DBS samples. This can result in better sequencing coverage of low-density *P. falciparum* infections. This higher sequencing coverage is necessary for robust population genomic analyses^7^. Using the SWGA method, we generated 15 whole genome sequences of *P. falciparum* from 15 dried blood spot samples. These DBS samples were collected from asymptomatic individuals of all ages during a cross sectional malaria prevalence survey conducted on five islands on the archipelago in 2018. This is the first WGS data from *P. falciparum* parasites on these islands. These data provide insights into the genetic variation and population dynamics of the *P. falciparum* parasites, including genomic markers associated with antimalarial drug resistance. The genomic variation within the Bijagós Archipelago is contextualised with approximately 800 isolates from other regions in Africa, using publicly available WGS data^8^. Importantly, these data provide a baseline assessment of genomic variation in 2018, prior to the subsequent implementation of SMC and MDA programmes in the region.

## Results

### Whole genome sequence data

The filtering steps for *P. falciparum* isolates for inclusion in genomic analyses can be seen in Supplementary Fig. 4. DNA was extracted from 2500 dried blood spots. qPCR was then used to test for the presence of *P. falciparum* in the extracted DNA. This identified 145 samples which were positive for *P. falciparum* with a CT value < 30*. P. falciparum* DNA was successfully amplified using Selective Whole Genome Amplification to sufficient levels for whole genome sequencing in 48 samples. Downstream filtering for high-quality whole genome sequence data resulted in 15 samples being carried forwards for bioinformatic analysis (please refer to the Methods section for additional detail).

A pairwise nucleotide matrix of 1,326,629 filtered SNPs was generated using whole genome sequences from 795 *P. falciparum* isolates, collected from 10 countries across Africa (Supplementary Table 1). This included isolates from Central Africa (Gabon, n = 57), West Africa (Ivory Coast n = 70, The Gambia n = 148, Bijagós Archipelago Guinea-Bissau (n = 15), East Africa (Kenya n = 138, Uganda n = 17), Southeast Africa (Malawi n = 149, Madagascar n = 24), South Central Africa (DRC n = 150) and Horn of Africa (Eritrea n = 27). The F_WS_ metric was used to investigate within-sample diversity. Most samples appeared monoclonal, with “high” F_WS_ estimates of ≥ 0.95^9^ (Supplementary Table 2). The samples from the Bijagós had a mean F_WS_ of 0.965, with 86.7% monoclonality (F_WS_ ≥ 0.95), which was higher than isolates from other countries, including The Gambia and Ivory Coast, which exhibited 67.6% and 61.4% monoclonality respectively. This high F_WS_ score indicates low multiplicity of infection within samples from the Bijagós. This may be due to low transmission intensity overall, or be because people generally live in small, more isolated communities, and supports the assumption that our population analyses are robust.

### *P. falciparum* isolates from the Bijagós Archipelago cluster within West Africa

**Figure 1:**
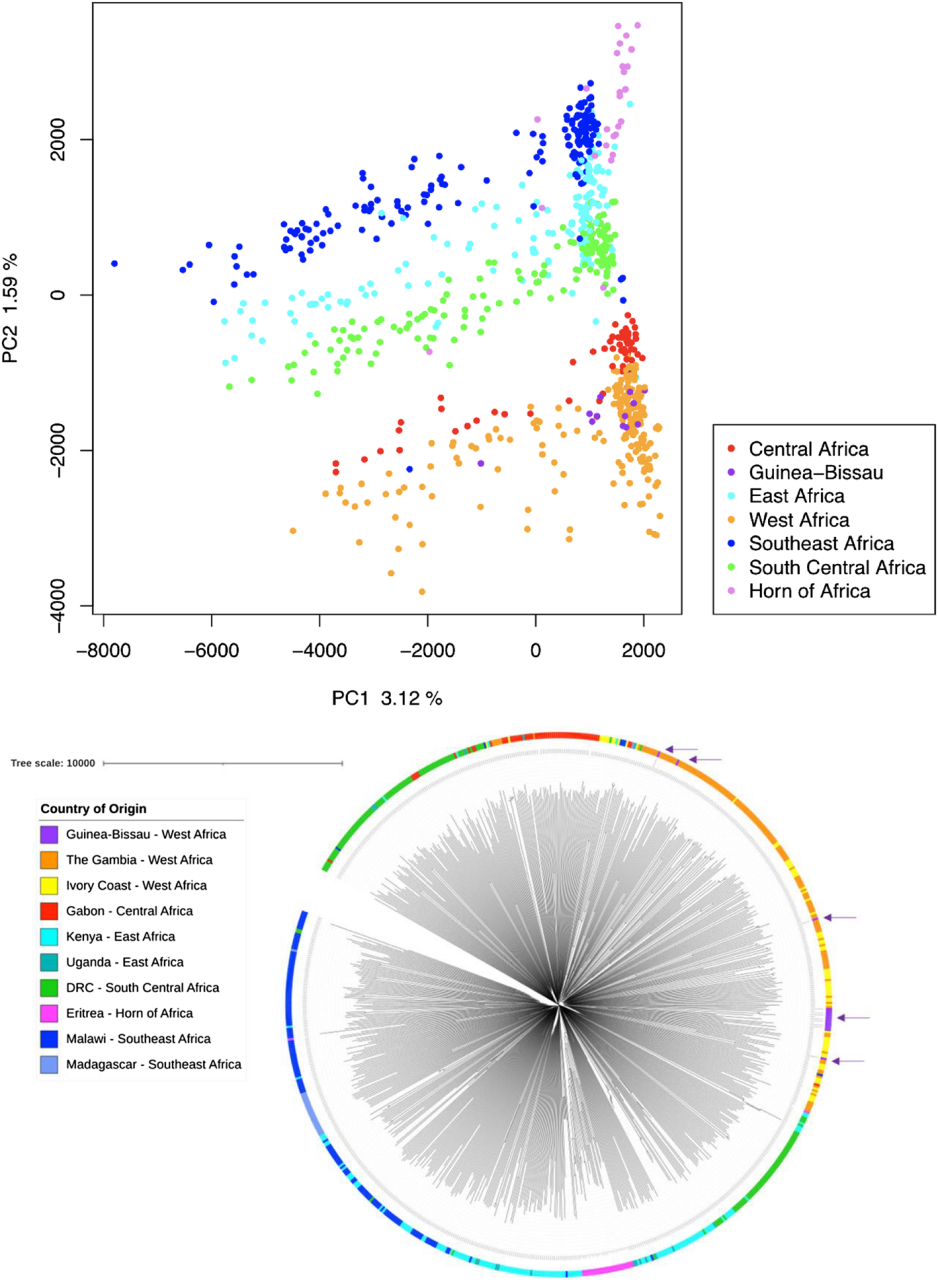
Population structure analysis: (top) Principal component analysis demonstrating that the isolates from the Bijagós (in purple) cluster with other isolates from West Africa (in orange and yellow). Generated using pairwise genetic distance matrices containing 1,326,629 SNPs from 795 isolates. (bottom) Neighbour-Joining tree for the samples and SNPs described above. Isolates were sampled from Central Africa (Gabon, n = 57), West Africa (Ivory Coast n = 70, The Gambia n = 148, Bijagós Archipelago Guinea-Bissau (n = 15), East Africa (Kenya n = 138, Uganda n = 17), Southeast Africa (Malawi n = 149, Madagascar n = 24), South Central Africa (DRC n = 150) and Horn of Africa (Eritrea n = 27). Bijagós isolates (indicated with purple arrows) are found interspersed with samples from West Africa (orange and yellow).

Principal component analysis (PCA) was conducted using the previously described pairwise genetic distance matrices to investigate the population structure of the *P. falciparum* isolates. Isolates from the Bijagós Archipelago were found to cluster with samples from West Africa when compared to other regions of Africa (Fig. 1). A SNP based Neighbour-Joining tree was generated to further investigate where the Bijagós *P. falciparum* isolates were situated phylogeographically. The samples from the Bijagós (labelled as Guinea-Bissau in purple), clustered with other West African countries. The samples from the Bijagós, The Gambia and Ivory Coast appear to be mixed and closely related, without forming distinct clusters. This suggests a high degree of mixing between parasites from the Bijagós with those from neighbouring West African countries, reflecting a high degree of regional transmission of *P. falciparum* across West African borders, which has been previously reported^10^.

### Population differentiation

Population differentiation due to genetic structure was measured using Fixation index (FST) statistics^11^, where SNPs with values of 1 indicate perfect differentiation between populations. SNPs that differentiated the Bijagós Archipelago from other African countries or regions were identified. SNPs with a threshold of F_ST_ > 0.75, F_ST_ > 0.85, and F_ST_ > 0.9 were identified to investigate patterns of differentiation between isolates from the Bijagós and the rest of Africa.

**Figure 2:**
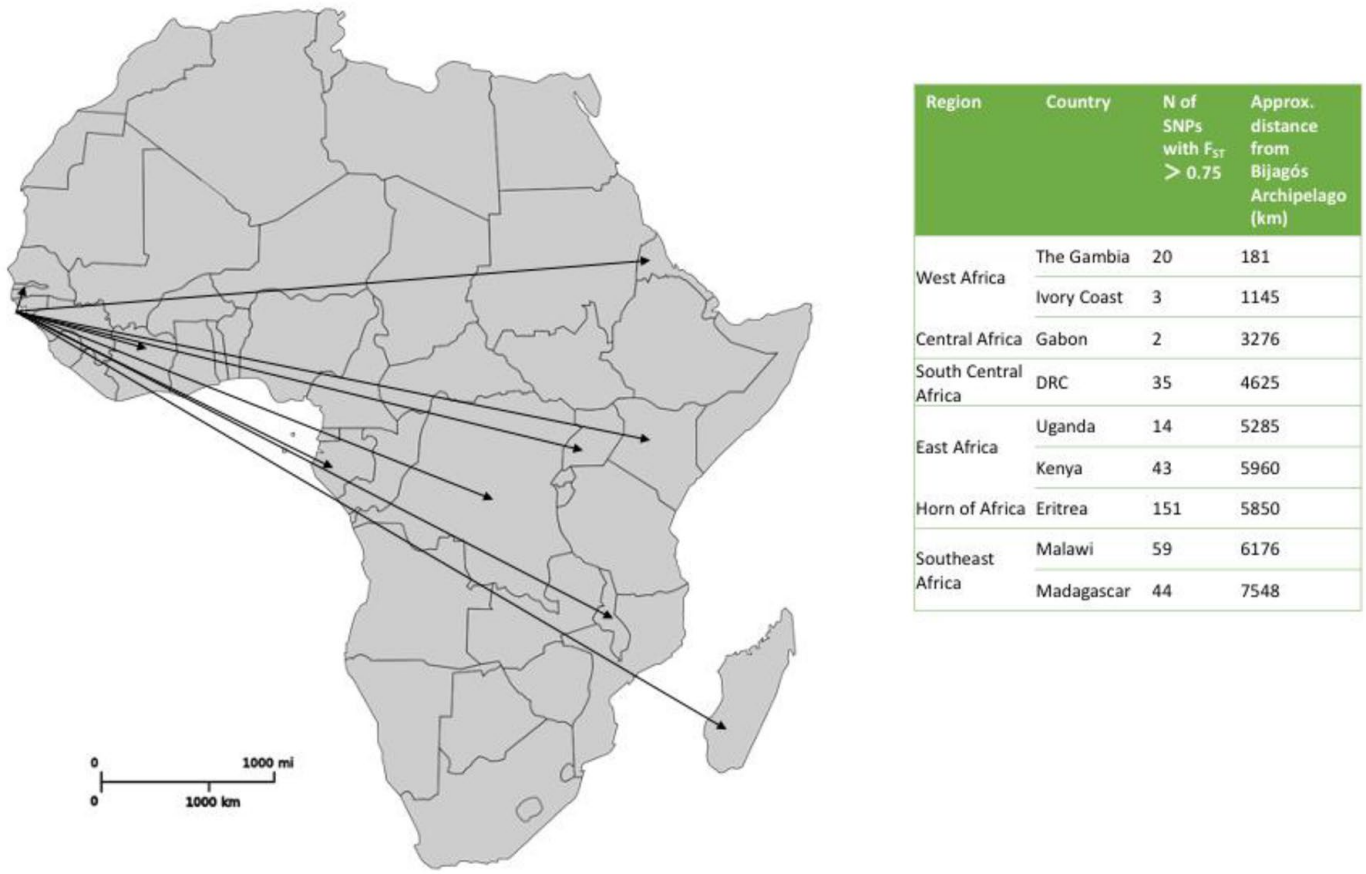
Map showing the proximity of the Bijagós Archipelago to the other countries within this dataset, and the number of SNPs with F_ST_ > 0.75 for each country comparison.

The isolates from the Bijagós were compared separately to each of the other countries within the dataset. The number of SNPs with F_ST_ > 0.75 for each country comparison are described in Fig. 2. There was an expected trend towards greater SNP differences with increased geographical distance. The largest degree of population differentiation was found between the Bijagós and Eritrea, with 151 SNPs with F_ST_ > 0.75.

SNPs with an F_ST_ > 0.85 were identified by comparing isolates from the Bijagós with those from other countries in West Africa (N = 233), East Africa (N = 155), Southeast Africa (N = 172), South Central Africa (N = 150), and the Horn of Africa (N = 27), respectively (Table 1). When comparing the isolates from the Bijagós with samples from other African regions, there were 15 SNPs with F_ST_ > 0.9. This included 2 SNPs when comparing isolates from the Bijagós with East Africa, and 13 SNPs when comparing with the Horn of Africa (Table 1). SNPs with F_ST_ > 0.9 included those in the rhoptry-associated protein 1, RAP1 (PF3D7_1410400), and reticulocyte binding protein homologue 1 (PfRh) (PF3D7_0402300). The RAP1 protein is part of the rhoptry-associated protein (RAP) complex, which is important for parasitophorous vacuole membrane structure and intraerythrocytic parasite growth^12^. The PfRh protein is part of the reticulocyte binding-like (RBL) family, binds to erythrocytes and plays a key role in merozoite invasion^13^.

**Table 1:** Genetic markers of population differentiation between the isolates from the Bijagós Archipelago and isolates from countries in the other African regions, with F_ST_ > 0.85. SNPs with F_ST_ > 0.9 have been highlighted in bold.

Chr	Position	Gene	F_ST_ value	% Alternate allele – Bijagós population	% Alternate allele – Population 2	Population 2
10	331,746	PF3D7_1008100: PHD finger protein PHD1	0.892	50.0	0.5	West Africa
12	1,265,899	PF3D7_1230800: pre-mRNA-splicing regulator WTAP, putative	0.864	40.0	0	West Africa
6	697,009	PF3D7_0616800 malate:quinone oxidoreductase	0.854	0	91.0	East Africa
6	1,110,905	PF3D7_0627700 transportin	0.855	50	0	East Africa
6	1,115,439	PF3D7_0627800 acetyl-CoA synthetase	0.888	64.3	0.6	East Africa
**7**	**732,862**	eukaryotic translation initiation factor 3 subunit I, putative	**0.922**	**83.3**	**0.6**	**East Africa**
9	1,178,903	high molecular weight rhoptry protein 2	0.873	92.9	5.2	East Africa
9	1,402,365	PF3D7_0935500 *Plasmodium* exported protein, unknown function	0.869	73.3	1.6	East Africa
10	331,746	PF3D7_1008100: PHD finger protein PHD1	0.853	50.0	0.7	East Africa
10	658,448	serine/threonine protein kinase, FIKK family	0.865	81.8	3.9	East Africa
11	1,376,369	PF3D7_1135100: protein phosphatase PPM8, putative	0.870	13.3	94.2	East Africa
**14**	**421,792**	**rhoptry-associated protein 1**	**0.914**	**21.4**	**98.7**	**East Africa**
6	1,115,439	PF3D7_0627800 acetyl-CoA synthetase	0.887	64.3	0.6	Southeast Africa
7	732,862	eukaryotic translation initiation factor 3 subunit I, putative	0.893	83.3	1.7	Southeast Africa
10	331,746	PF3D7_1008100: PHD finger protein PHD1	0.899	50.0	0	Southeast Africa
12	1,265,899	PF3D7_1230800: pre-mRNA-splicing regulator WTAP, putative	0.886	40.0	0	Southeast Africa
12	1,265,899	PF3D7_1230800: pre-mRNA-splicing regulator WTAP, putative	0.882	12.5	0	South Central Africa
**2**	318,919 and **319,674**	**PF3D7_0207900** **serine repeat antigen 2**	**0.929**	0	**94.4**	**Horn of Africa**
**2**	**367,271**	**6-cysteine protein P230p**	**0.934**	**0**	**94.7**	**Horn of Africa**
3	204,230	PF3D7_0304100 inner membrane complex protein 1e, putative	0.865	0	89.5	Horn of Africa
3	729,186	PF3D7_0317700: CPSF (cleavage and polyadenylation specific factor), subunit A, putative	0.859	0	87.5	Horn of Africa
**4**	**138,554** and 138,623	**reticulocyte binding protein homologue 1**	**0.942**	**0**	**95.8**	**Horn of Africa**
5	608,869	PF3D7_0514600: ribose-5-phosphate isomerase, putative	0.888	0	91.3	Horn of Africa
5	1,042,252	PF3D7_0525100 acyl-CoA synthetase	0.865	78.6	0	Horn of Africa
7	732,865	eukaryotic translation initiation factor 3 subunit I, putative	0.882	100	8.7	Horn of Africa
**7**	**894,384**	**PF3D7_0720700: phosphoinositide-binding protein PX1**	**0.923**	**100**	**3.7**	**Horn of Africa**
8	585,854	PF3D7_0811600: conserved protein, unknown function	0.895	0	92.6	Horn of Africa
**8**	**1,031,401**	**histone acetyltransferase GCN5**	**0.948**	**92.9**	**0**	**Horn of Africa**
**8**	**1,056,829**	**conserved *Plasmodium *protein, unknown function**	**0.948**	**100**	**3.7**	**Horn of Africa**
8	1,239,302	PF3D7_0828800: GPI-anchored micronemal antigen	0.879	0	88.9	Horn of Africa
**8**	**1,345,148**	***Plasmodium* exported protein, unknown function**	**1.000**	**100**	**0**	**Horn of Africa**
9	138,846	PF3D7_0801900: lysine-specific histone demethylase, putative	0.877	0	88.5	Horn of Africa
9	778,075	PF3D7_0918900: gamma-glutamylcysteine synthetase	0.870	0	90.5	Horn of Africa
**9**	**1,178,903 and 1,179,069**	**high molecular weight rhoptry protein 2**	**0.970**	**92.9**	**0**	**Horn of Africa**
**9**	**1,316,936**	**PF3D7_0933100: conserved** ***Plasmodium*** **protein, unknown function**	**0.925**	**0**	**92.3**	**Horn of Africa**
**9**	**1,406,576**	**PF3D7_0935600: gametocytogenesis-implicated protein**	**0.916**	**100**	**4.3**	**Horn of Africa**
9	1,407,002	PF3D7_0935600: gametocytogenesis-implicated protein	0.884	0	90.5	Horn of Africa
9	1,437,289 and 1,438,135	PF3D7_0936300: ring-exported protein 3	0.884	92.3	3.8	Horn of Africa
**10**	**658,448**	**serine/threonine protein kinase, FIKK family**	**0.908**	**81.8**	**0**	**Horn of Africa**
11	354,078	PF3D7_1108000: IWS1-like protein, putative	0.890	0	91.3	Horn of Africa
11	812,065	PF3D7_1121400: WD repeat-containing protein, putative	0.894	0	92.0	Horn of Africa
11	1,050,233	PF3D7_1126800: alternative splicing factor SR-MG, putative	0.862	0	87.0	Horn of Africa
11	1,376,369	PF3D7_1135100: protein phosphatase PPM8, putative	0.894	13.3	100	Horn of Africa
**11**	**1,507,877**	**guanylyl cyclase alpha**	**0.925**	**0**	**92.3**	**Horn of Africa**
**12**	**666,002**	**perforin-like protein 2**	**0.943**	**0**	**95.7**	**Horn of Africa**
12	1,998,804	PF3D7_1248700: conserved protein, unknown function	0.876	0	88.5	Horn of Africa
13	2,107,756	PF3D7_1352700: intron-binding protein aquarius, putative	0.851	0	88.9	Horn of Africa
14	812,013	PF3D7_1419400: conserved *Plasmodium* membrane protein, unknown function	0.878	0	90.5	Horn of Africa
14	1,227,066	PF3D7_1431200: OST-HTH associated domain protein, putative	0.875	6.7	95.2	Horn of Africa
14	1,989,372	PF3D7_1448500: conserved *Plasmodium* protein, unknown function	0.888	7.7	96.3	Horn of Africa
14	2,004,493	PF3D7_1449000: gamete egress and sporozoite traversal protein, putative	0.899	13.3	100	Horn of Africa
14	2,340,921	PF3D7_1457100: conserved *Plasmodium* protein, unknown function	0.876	0	88.5	Horn of Africa
14	2,343,679	PF3D7_1457200: thioredoxin 1	0.879	0	88.9	Horn of Africa
14	2,357,358	PF3D7_1457400: conserved *Plasmodium* protein, unknown function	0.897	0	92.6	Horn of Africa
14	2,364,543	PF3D7_1457600: conserved *Plasmodium* protein, unknown function	0.869	0	92.6	Horn of Africa
14	2,364,847	PF3D7_1457600: conserved *Plasmodium* protein, unknown function	0.854	0	88.9	Horn of Africa
14	2,367,254	PF3D7_1457700: large ribosomal subunit nuclear export factor, putative	0.851	0	88.5	Horn of Africa
14	2,469,318	PF3D7_1460500: conserved *Plasmodium* protein, unknown function	0.894	0	91.7	Horn of Africa
14	2,535,998	PF3D7_1462400: conserved *Plasmodium* protein, unknown function	0.899	0	92.6	Horn of Africa
14	2,845,773	PF3D7_1469400: nucleoside transporter 3, putative	0.859	0	87.5	Horn of Africa

Only SNPs where all calls in the Bijagós population were either reference, alternate, or mixed, were included.

Sample sizes: Bijagós Archipelago N = 15, West Africa N = 233, East Africa N = 155, Southeast Africa N = 172, South Central Africa N = 150, Horn of Africa N = 27.

### Genetic relatedness

DNA segments that are identical between individuals and have been inherited from a common ancestor without recombination are termed identical by descent (IBD)^14^. IBD analysis was conducted to infer pairwise identity by descent between *Plasmodium* isolates within populations. Genomic relatedness was determined by analysing the proportion, or fraction, of the genome that was IBD between the *P. falciparum* isolates. Cumulative IBD across the genome was calculated using sliding window analysis for each population using windows of 10,000 bases (Supplementary Table 3). Isolates from the Bijagós exhibited median IBD fractions of 0.04, ranging between 0.001 and 0.217. This was slightly higher than the other countries in West Africa; Ivory Coast had a median IBD of 0.003, while The Gambia had a median IBD of 0.011. Higher IBD within a population indicates that a higher proportion of alleles at the same locus were inherited from a common ancestor. The higher IBD score for the Bijagós isolates suggests reduced outcrossing within the population, in comparison with other West African countries, which is likely to reflect their isolated island status.

### Genomic regions under recent positive selection

WGS data was analysed for signatures of recent positive selection. Single population iHS and cross-population XP-EHH metrics were calculated, which are statistics derived from extended haplotype homozygosity. Within the isolates from the Bijagós, there were seven SNPs which exhibited significantly high iHS scores, where (− log_10_[1 – 2 | Φ_iHS_ – 0.5 |]) > 4.0 (Supplementary Table 4). These SNPs were located within two surface-associated interspersed (SURFIN) pseudogenes, SURFIN 1.2 and SURFIN 13.1. Cross-population analysis was conducted between isolates from the Bijagós and other regional populations within Africa. This analysis identified seven candidate regions under directional selection based on XP-EHH values (Supplementary Table 5). When comparing isolates from the Bijagós with those from all other African regions, there were four common candidate regions under directional selection. These regions encoded cytoadherence linked asexual proteins 3.1 and 3.2, the ABC transporter B family member 4 (putative), and the CX3CL1-binding protein 2. When comparing isolates from the Bijagós to those from Central Africa, South Central Africa, Southeast Africa and West Africa, there were two additional common candidate regions under directional selection. These regions encode the 26S protease regulatory subunit 8 (putative) and a conserved *Plasmodium* protein of unknown function (PF3D7_1248700). When comparing isolates from the Bijagós with isolates from Southeast Africa and West Africa, two additional common candidate regions were identified, which encode merozoite surface protein 3 and methionine tRNA ligase. When comparing with East Africa, one additional candidate region was found, encoding Plasmepsin X. When comparing with South Central Africa, two additional candidate regions were identified, encoding the 26S protease regulatory subunit 6A (putative), and structural maintenance of chromosomes protein 1 (putative) (Supplementary Table 5).

### Population admixture

**Figure 3:**
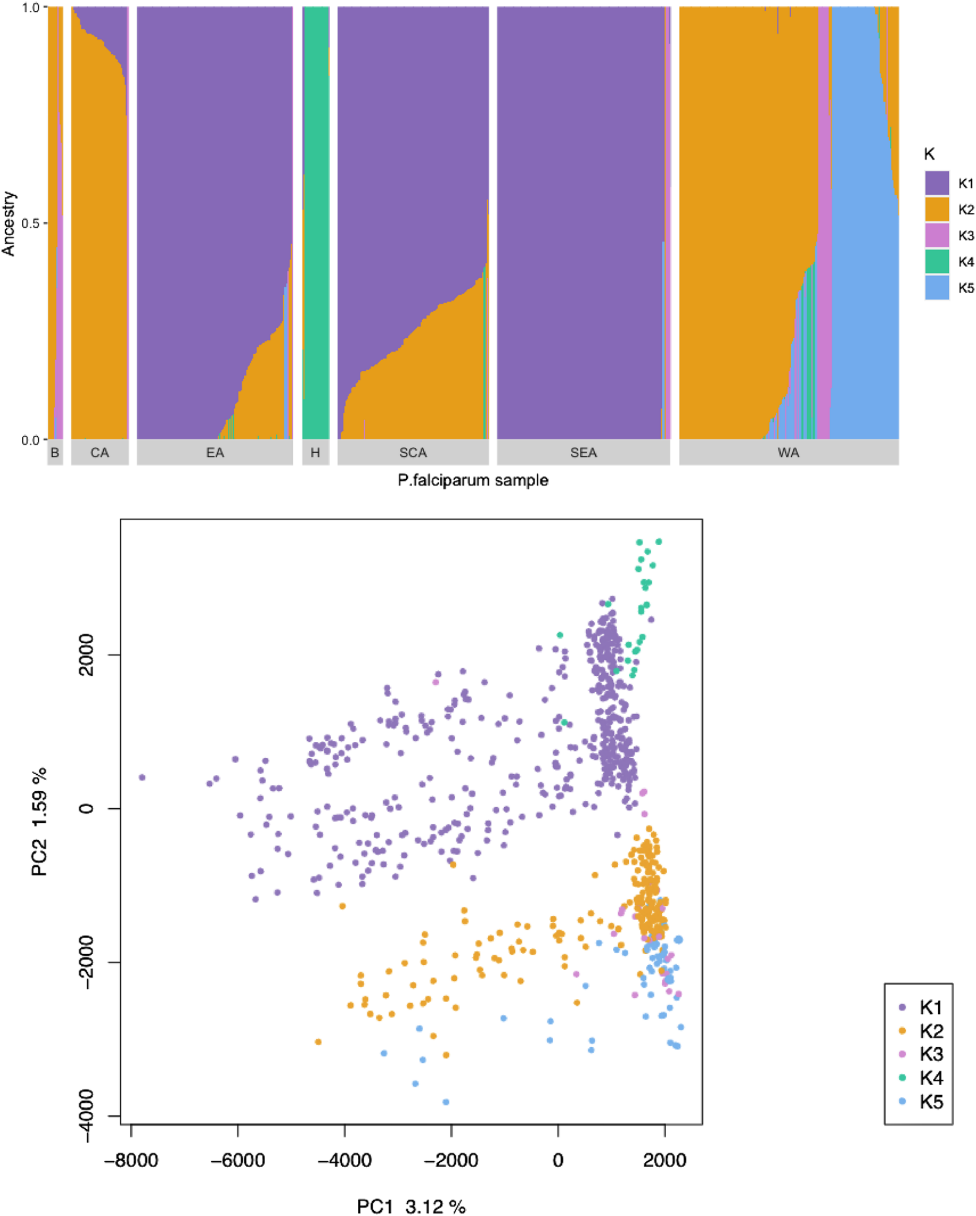
(top) Ancestry analysis of *P. falciparum* isolates from (B) the Bijagós Archipelago, (CA) Central Africa, (EA) East Africa, (H) Horn of Africa, (SCA) South Central Africa, (SEA) Southeast Africa and (WA) West Africa. Individual isolates are plotted along the x-axis, with ancestry coefficients on the y-axis. The number of ancestral populations, K, used in this analysis was K = 5. (bottom) Principal component analysis generated using pairwise genetic distance matrices. This is coloured by the maximum K value for each isolate.

Admixture analysis is a method of investigating the geographical origins of samples, based on their ancestry. Admixture analysis was conducted on 387,395 SNPs using ADMIXTURE software. Using a cross-validation approach, five ancestral populations were inferred (K value = 5). Figure 3 (top) shows these five ancestral populations (K = 1 to K = 5) as different colours. The figure includes each individual *P. falciparum* isolate along the x-axis, and the ancestral populations of each isolate along the y-axis, represented by colour. This analysis suggested that isolates from the Bijagós contained high proportions of ancestral genome fragments associated with West African *P. falciparum* populations (Fig. 3).

PCA was carried out using the previously described pairwise genetic distance matrix containing 1,326,629 SNPs from 795 isolates. Each *P. falciparum* isolate on the PCA was coloured according to its dominant ancestral population (of K = 1 to K = 5). This is shown in Fig. 3 (bottom). The overlapping of dominant ancestry reinforced high proportions of shared ancestral genome fragments for the samples across Africa (Fig. 3).

### Identification of mutations associated with drug resistance

Mutations associated with resistance to antimalarial drugs were analysed using the WGS data from the Bijagós isolates (n = 15) and isolates from West Africa (n = 218). Comparison of the prevalence of drug resistance mutations between these populations was carried out to understand similarities and differences between these isolates (Table 2).

**Table 2:** Frequency of mutations associated with resistance to different anti-malarial drugs, from Bijagós *P. falciparum* isolates (n = 15, sample collection in 2018) and *P. falciparum* isolates from West Africa (Total n = 218, Ivory Coast n = 70—sample collection in 2013, The Gambia n = 148—sample collection in 2008 and 2014).

Gene	Position	Mut	Ref allele	Alt allele	n/N mutation Bijagós	n/N mutation W. Africa	% Mut in Bijagós	% Mut in W. Africa	*P* value (X^2^)
*pfcrt*	403,625	K76T	A	C	4/14	112/218	28.6	51.4	0.098
*pfdhfr*	748,239	N51I	A	T	14/14	167/218	100	76.6	0.040
*pfdhfr*	748,262	C59R	T	C	13/14	169/218	92.9	77.5	0.158
*pfdhfr*	748,410	S108N	G	A	14/14	176/218	100	81.1	0.070
*pfdhps*	549,993	K540E	A	G	0/15	0/218	0	0	-
*pfdhps*	549,685	A437G	C	G	9/15	162/218	60.0	74.3	0.225
*pfdhps*	550,117	A581G	C	G	0/15	2/218	0	0.9	0.709
*pfdhps*	549,681	S436A	T	G/C	3/15	64/218	20.0	29.4	0.439
*pfmdr1*	958,145	N86Y	A	T	1/13	34/218	7.7	15.6	0.440
*pfmdr1*	958,440	Y184F	A	T	7/13	131/218	53.8	60.1	0.656
*pfmdr1*	961,625	D1246Y	G	T	0/13	2/218	0	0.9	0.729
*pfkelch13*	1,725,626	N458Y	T	A/C	0/14	0/218	0	0	-
*pfexo*	2,504,560	E415G	A	G	0/13	0/218	0	0	-
*pfap2µ*	718,433	S160N/T	G	A/C	0/14	12/218	0	5.5	0.367

The K76T mutation, associated with resistance to chloroquine^15^, was found at a slightly lower frequency (28.6%) in isolates from the Bijagós compared to isolates from The Gambia and Ivory Coast in mainland West Africa (51.4%) (non-significant *P* > 0.05). Overall, PfDHFR mutations associated with resistance to pyrimethamine^16^ were found at higher prevalence in isolates from the Bijagós than those from mainland West Africa. N51I was found at significantly higher prevalence in the Bijagós (100%) than in mainland West Africa (76.6%) (*P* < 0.05). S108N was found at 100% prevalence in the Bijagós compared to 81.1% in mainland West Africa (non-significant *P* > 0.05). C59R was found at 92.9% prevalence compared to 77.5% in mainland West Africa (non-significant *P* > 0.05). Of the 15 Bijagós samples sequenced, one was a double mutant with N51I-S108N, and 13 were triple mutants with N51I-C59R-S108N. The PfDHPS A437G mutation, associated with resistance to sulphadoxine^17^, was found in 60% of isolates from the Bijagós. K540E was not found in isolates from the Bijagós or mainland West Africa. A581G was not found in isolates from the Bijagós but was found at a prevalence of 0.9% in isolates from mainland West Africa (non-significant difference *P* > 0.05). Of the 15 Bijagós samples sequenced, two were double mutant with S436A-A437G.

PfMDR1 mutations N86Y and Y184F, associated with resistance to chloroquine^18,19^, were found in the Bijagós isolates at frequencies of 7.7% and 53.8%, respectively. The prevalence of N86Y and Y184F was slightly higher in isolates from mainland West Africa, at 15.6% and 60.1%, respectively (non-significant difference *P* > 0.05). The mutation D1246Y, also associated with chloroquine resistance^18,19^, was not found in isolates from the Bijagós, but was found in 0.9% of isolates from mainland West Africa (non-significant difference *P* > 0.05). The N458Y mutation in PfKELCH13*,* associated with artemisinin partial resistance^20^, was not found in isolates from the Bijagós or mainland West Africa. There was not sufficient genomic coverage within the Bijagós sample WGS data of other PfKELCH13 marker positions to report these reliably. Mutations which could not be reported on are listed in Supplementary Table 7. The PfEXO E415G mutation, associated with resistance to piperaquine, and the PfAP2µ S160N/T mutation, which may be associated with artemisinin-resistance, were not found in either population^21^.

## Discussion

This study presents *Plasmodium falciparum* sequence data from the Bijagós Archipelago, Guinea-Bissau. Investigating the genomic architecture of malaria parasites in endemic countries increases the understanding of parasite genetic variation and population dynamics. This includes the prevalence of genomic mutations associated with drug resistance. Knowledge of drug resistance markers and population dynamics can assist in malaria control and surveillance, which are key to reducing the global malaria burden in line with the World Health Organization (WHO) Global Technical Strategy^1^. Genomic surveillance of drug resistance markers is particularly important considering the likely increase in the mass use of antimalarials following recently expanded WHO recommendations^2^.

The Bijagós Archipelago is a region with low-moderate malaria transmission intensity, exhibiting stable and seasonal malaria cases. Many malaria cases in the region are asymptomatic, which presents a difficult challenge to control programmes, as asymptomatic cases sustain a reservoir of infections^22^. Consequently, this reservoir is capable of continually driving malaria transmission, which makes eliminating malaria in this area extremely difficult^23,24^. Furthermore, drug resistance is hypothesised to spread faster in low transmission settings than in high transmission settings due to a higher proportion of infections being exposed to antimalarial drugs. This increases the probability of treatment failure and the subsequent transmission of resistant parasites in the population^25^.

The data analyses presented in this study characterise asymptomatic malaria infections. These infections are more difficult to study due to the low quantities of parasite DNA in clinical samples, particularly in dried blood spots (DBS). This means that despite the importance of understanding asymptomatic cases, most research into genomic variation and drug resistance is conducted on parasites from symptomatic malaria infections. This study made use of Selective Whole Genome Amplification (SWGA) to amplify parasite DNA from isolates collected using DBS, from asymptomatic infections within the Bijagós^7^. These advances in molecular methods, along with improved Next Generation Sequencing technology, allow for feasible routine surveillance of parasite genetic variation and drug resistance markers from dried blood spot samples, which can be collected during routine cross-sectional surveys.

Following successful amplification of low-density asymptomatic *P. falciparum* infections obtained in the Bijagós, population analyses were conducted. The Bijagós *P. falciparum* genomic variation was then contextualised with the wider African *P. falciparum* population. Principal component analysis (PCA) indicated that the malaria samples from the Bijagós cluster with samples from other countries in West Africa, including The Gambia and Ivory Coast. A relatively low percentage of the variance between malaria samples from different countries was explained within the PCA. This, along with the absence of SNPs with F_ST_ = 1, reflects the high degree of mixing between *P. falciparum* isolates in the Bijagós Archipelago and those on the mainland. Despite this relatively small sample size of 15 from the Bijagós, these results corroborate the high degree of mixing between *P. falciparum* isolates demonstrated across the African continent^10^. The samples from the Bijagós had a mean F_WS_ of 0.965, with 86.7% monoclonality (with Fws ≥ 0.95). This was the highest percentage of monoclonality when compared with samples from other countries across the African continent. The high percentage of monoclonal infections may be due to people in the Bijagós living in small, relatively isolated, communities, which could reduce the likelihood of simultaneous infection with parasites of different genotypes. This monoclonality may also reflect the low to moderate transmission intensity of *P. falciparum* in the Bijagós. Despite the high degree of population mixing, identity by descent analysis revealed that isolates from the Bijagós had slightly higher IBD fractions than isolates from the other West African countries, which may reflect their more isolated island status. Admixture analysis gave further insight into the ancestral origins of the Bijagós Archipelago isolates amongst African populations, suggesting Bijagós isolates obtained large proportions of their ancestral genome fragments from West African *P. falciparum* lineages.

Fixation index (F_ST_) analyses were used to measure population differentiation due to genetic structure. F_ST_ values range from 0 to 1, where a value of 0 for a particular SNP indicates that two populations have no difference in allele frequency, whereas a value of 1 indicates complete differentiation in allele frequency between the two populations ^11^. Therefore, a value of 0 would indicate a high degree of population mixing. Whereas, higher F_ST_ values at a particular SNP indicate greater population differentiation. Fifteen SNPs with a high F_ST_ > 0.9 were identified when comparing isolates from the Bijagós with those from other African regions. The greatest amount of population differentiation was found between samples from the Bijagós and Eritrea. Interestingly, SNPs in the Bijagós samples were not fixed at either allele for many of the loci with high F_ST_ values, indicating that the population is in transition for many of these alleles. Overall, there were no SNPs with F_ST_ = 1 in any of the regional comparisons. This indicates that there were no SNPs specific to the Bijagós parasite population. It is likely that a larger sample size would be needed to reveal these SNPs with F_ST_ = 1, which could then be used to generate a molecular barcode for the *P. falciparum* samples from the Bijagós able to predict geographic origin^26^.

Regions under selective pressure included seven loci within the surface-associated interspersed (SURFIN) pseudogenes, SURFIN 1.2 and SURFIN 13.1. SURFINs are variable erythrocyte surface antigens and are targets of naturally acquired immunity against malaria^27^. SURFIN 1.2 has been associated with chloroquine sensitivity^27^, and it has been suggested that both SURFIN 1.2 and SURFIN 13.1 are involved in cell surface adhesion^27,28^. However, additional phenotypic experimentation is required to confirm the role of these two pseudogenes. Cross-population analysis identified seven candidate regions under directional selection based on XP-EHH values. These candidate regions included genes encoding Plasmepsin X (PMX), the ABC transporter B family member 4 (ABCB4), and *P. falciparum* merozoite surface protein 3 (PfMSP3). Plasmepsin X is essential for asexual parasite development and is considered a multi-stage antimalarial target^29^. ABCB4 is a member of the ABC (ATP-binding cassette) B family. This is a group of transport proteins which includes the PfMDR1 protein, and polymorphisms in the ABC transporters have been involved in anti-malarial drug resistance^30^. PfMSP3 is a merozoite surface protein and is a potential vaccine candidate^31^.

This study reported on genomic markers associated with drug resistance in the Bijagós Archipelago. Antimalarial treatments administered on the islands include artemether-lumefantrine and IPTp with sulphadoxine-pyrimethamine (SP). Intravenous artesunate or quinine are only used in severe, or complicated, cases. The PfCRT K76T mutation, associated with resistance to chloroquine, had a frequency of 28.6% on the islands, despite chloroquine not being included in malaria treatment policy since 2006 in Guinea-Bissau. This was a slightly lower prevalence than observed in isolates from mainland West Africa, but this difference was not significant (*P* > 0.05). Persistence of the K76T mutation may indicate that it has not yet been lost due to fitness-cost. The PfDHFR mutations associated with resistance to pyrimethamine, N51I and S108N, were found to be fixed (100% prevalence) in the Bijagós isolates, and C59R was found close to fixation at 92.9% prevalence. These mutations were found at a higher prevalence in the Bijagós isolates than in the isolates from mainland West Africa, with the N51I mutation at significantly higher prevalence in the Bijagós (*P* = 0.04). This difference may be partially explained by the collection years of these isolates, as isolates from the Bijagós were collected in 2018, whereas isolates from mainland West Africa were collected earlier, between 2008 and 2014. Therefore, the Bijagós isolates were collected following additional years of selection pressure from the use of SP in the region (Supplementary Table 6**)**. Thirteen of the Bijagós isolates sequenced exhibited the triple mutant N51I-C59R-S108N haplotype. In addition, the PfDHPS A437G and S436A mutations, associated with resistance to sulphadoxine, were found at 60% and 20% prevalence, respectively. Although this is a small sample set of 15 isolates, the presence of mutations associated with resistance to SP warrants further investigation with a larger sample size. Encouragingly, the PfEXO E415G mutation associated with piperaquine resistance, and the PfAP2µ S160N/T mutation, which may be associated with artemisinin-resistance, were not found in the Bijagós isolates. Limited sequencing coverage of the PfKELCH13 gene made investigating the majority of validated or candidate resistance mutations in this gene unreliable. However, there was sufficient coverage of the N458Y genome position to identify reliably that this mutation was absent in the Bijagós isolates. The artemisinin-combination therapy dihydroartemisinin-piperaquine (DHA-PPQ) was not used on the islands prior to this study. However, there has since been MDA with DHA-PPQ across the archipelago.

Despite the limitations in generating sequence data from asymptomatic isolates collected using DBS, this study reported high quality whole genome sequence data for 15 Bijagós isolates. This is a small sample size which has limitations for generalisation. However, the population genomic analyses results are consistent with those from neighbouring African countries, indicating that these results are robust. Furthermore, this study further demonstrates the capacity of SWGA methods in generating WGS data from low-density parasite concentrations. This dataset forms the only dataset of WGS data for the Bijagós. Therefore, it forms an important baseline in the context of evaluating subsequent MDA and SMC interventions that have been implemented in the region. The Bijagós WGS data was contextualised with publicly available WGS sequence data from isolates across Africa. Information about the symptomatic or asymptomatic status of these infections is not publicly available, so comparisons concerning patient symptom status cannot be made using this dataset. Additional sequencing of *P. falciparum* isolates is required to reveal a more comprehensive picture of regional dynamics, particularly concerning the prevalence of drug resistance markers. This should ideally include the analysis of samples collected from both asymptomatic and symptomatic infections, and across different years, to understand temporal changes in resistance marker frequency. High throughput, low-cost, methods, such as amplicon sequencing, have the potential to achieve this. In turn, this information can be used to inform programmatic decisions in malaria control, working towards malaria elimination.

## Materials and methods

### Dried blood spot collection

Dried blood spots were collected onto filter paper from residents of the Bijagós Archipelago during a cross-sectional survey in 2018. Dried blood spots were sampled as part of this mapping survey, regardless of whether the participant had symptoms of malaria.

This study was conducted in accordance with the Declaration of Helsinki and granted ethical approval by the Comite Nacional de Eticas de Saude (Guinea-Bissau) and the London School of Hygiene and Tropical Medicine Research Ethics Committee (UK). Written or thumbprint informed consent was obtained from all study participants.

### DNA extraction and qPCR for malaria positivity

Following collection, dried blood spots were punched for DNA extraction using 3 mm diameter punches. Three punches were taken per sample for DNA extraction. DNA was extracted using the PureLink™ Pro 96 Genomic DNA Purification Kit (ThermoFisher), (cat No: K182104A). DNA extract was quantified for *Plasmodium falciparum* 18S gene using qPCR.

### *Plasmodium* genome amplification and sequencing

DNA extracts which had a qPCR CT value < 30 were tested using the Qubit for DNA concentration. Using this CT threshold, n = 145 samples were selected for further analysis. DNA concentration in the extracted dried blood spot samples was very low, of the range 0.011 ng/μl to 0.268 ng/μl. Selective whole genome amplification (SWGA) was used to amplify the *Plasmodium* DNA prior to sequencing, using a protocol adapted from Oyola et al.^7^. Each SWGA reaction used 30 µl of extracted DNA, which was combined with 5 µl of *phi29* DNA Polymerase Reaction Buffer (New England Biolabs), 0.5 µl of Recombinant Albumin (New England Biolabs), 0.5 µl of SWGA primers (250 mM) (Roche), 5 µl dNTPs (New England Biolabs) and 6 µl of water, to a total volume of 50 µl per reaction. SWGA reactions were then run in a thermocycler using the following programme steps: 35 °C for 5 min, 34 °C for 10 min, 33 °C for 15 min, 32 °C for 20 min, 31 °C for 25 min, 30 °C for 16 h, 65 °C for 10 min, and held at 4 °C. SWGA reactions increased the DNA concentration of samples to > 300 ng DNA in of the 48 of the 145 (33.1%) samples amplified.

### Whole genome sequencing

The SWGA product from these samples was purified using KAPA pure beads (Roche). Beads were equilibrated to room temperature and mixed with SWGA product at a 1:1 volume ratio. The KAPA pure beads Genomic DNA Purification protocol steps were then followed (https://rochesequencingstore.com/wp-content/uploads/2022/07/KAPA-Pure-Beads-Technical-Data-Sheet.pdf). DNA was then quantified using the Qubit dsDNA HS Kit (Thermo Fisher). Samples contained > 300 ng DNA and were sequenced at Eurofins Genomics using the Illumina Novaseq 6000, producing a minimum of 3.75 M paired reads (250 bp reads) per sample.

### Bioinformatics

FastQ files from WGS were used to produce VCF files for each sample. To do this, FastQ files were trimmed using *trimmomatic* software (version 0.39, using the parameters LEADING:3 TRAILING:3 SLIDINGWINDOW:4:20 MINLEN:36) to remove poor quality sequences^32^. The trimmed data was then aligned to the reference *P. falciparum* 3D7 genome (version 3) using *bwa-mem* software (version 0.7.17-r1188, default parameters), to produce a BAM file for each sample. The *samtools* (version 1.12) functions fixmate and markdup were applied to the resulting BAM files^33^. Following this, the *GATK’*s BaseRecalibrator and ApplyBQSR functions were applied for calibration and correction of the BAM files using the *P. falciparum* genetic crosses 1.0 dataset (http://www.malariagen.net/data/pf-crosses-1.0). SNPs and indels were called with *GATK’s* HaplotypeCaller (version 4.1.4.1) using the option -ERC GVCF^34^. Validated VCFs were imported into GenomicsDB using *GATK*’s function GenomicsDBImport, and a combined VCF was created using *GATK*’s GenotypeGVCFs function. This VCF file contains all the genotype calls for all the variant positions across all samples.

For further analysis, only variants in core regions of the genome were included and assigned a quality score using GATK's Variant Quality Score Recalibration (VQSR). VariantRecalibrator was run using the previously mentioned genetic crosses dataset as a training set with the following parameters used for SNPs: -an QD -an FS -an SOR -an DP -maxGaussians 8 and -mq-cap-for-logit-jitter-transform 70. Following this, GATK's ApplyVQSR was run using the parameter -truth-sensitivity-filter-level 99.0 to obtain a Variant Quality Score Log-Odds (VQSLOD). Variants with a VQSLOD score < 0, representing variants more likely to be false than true, were filtered out.

Additionally, isolates with more than 40% of SNPs missing genotype data (177/2129 isolates) were excluded from downstream analysis to ensure that samples which passed quality control had uniform sequencing coverage across the whole genome. SNPs were then processed to replace the genotype call in the VCF for a mixed call whenever the secondary MAF was at least 20% in a given SNP on each individual sample. A subset of only biallelic SNPs was also obtained from this VCF. The filtered VCF was then converted into a binary matrix consisting of genotypes (0 = reference allele; 1 = mutant allele; 0.5 = mixed allele) across all SNPs and isolates. The final matrix consisted of 1952 isolates and 1,326,629 high-quality SNPs. This bioinformatics pipeline is described in detail by Benavente et al.^35^. An annotated VCF of all extracted variants was made using *snpEff* software (version 5.1), which annotates the variants^36^. Fifteen of the Bijagós samples had sufficient quality data for downstream bioinformatic analysis, with over ~ 40% of the genome covered by > 5 reads.

### Population genomic analysis

Whole Genome Sequence data from the samples from the Bijagós were compared with publicly available data from mainland Guinea-Bissau and other African countries, through the MalariaGEN Pf3k project and ongoing LSHTM studies (https://www.malariagen.net/parasite/pf3k). This included whole genome sequences from 795 isolates, from 10 different countries across Africa (Supplementary Table 1). This included isolates from Central Africa (Gabon, n = 57), West Africa (Ivory Coast n = 70, The Gambia n = 148, Bijagós Archipelago Guinea-Bissau (n = 15), East Africa (Kenya n = 138, Uganda n = 17), Southeast Africa (Malawi n = 149, Madagascar n = 24), South Central Africa (DRC n = 150) and Horn of Africa (Eritrea n = 27). A binary matrix of pairwise genetic distances was constructed from this WGS data. To investigate population structure, Principal Component Analysis was conducted using the filtered bi-allelic nucleotide matrix, using the R package *amap* (version 0.8–18), and a neighbour-joining (N-J) tree was constructed using the R package *ape* (version 5.5). Neighbour-joining trees were visualised in iTOL^37^. Fixation index statistics (F_ST_) were calculated to infer nucleotide diversity and population differentiation using VCFtools (version 0.1.16, https://vcftools.github.io/documentation.html#fst). F_ST_ statistic outputs were only included in downstream analysis if a particular SNP had coverage in at least 2/3 of both of the total sample populations that were being compared.

The F_WS_ metric was used to quantify the extent of multiplicity of infection (MOI), by assessing within host diversity of *Plasmodium* in comparison to local population diversity^38^. This was calculated using the moimix R package (version 0.0.2.9001, https://bahlolab.github.io/moimix/)^39^. This indicated the frequency of inbreeding and outcrossing in the population. High F_WS_ (> 0.95) is highly indicative of monoclonality, whereas low F_WS_ indicates high levels of mixing between the parasites in the population, and subsequently a poorly defined population sub-structure.

IBD analysis was conducted using *hmmIBD* software (version 2.0.4) (default parameters). This software implements a hidden Markov model for detecting genomic regions that are identical by descent (IBD) for pairs of haploid samples^14^. Additional filtering steps were performed on the binary SNP matrix for IBD analysis: SNPs were selected with minor allele frequency > 0.01, samples were filtered for F_WS_ > 0.95 and mixed calls were filtered to alternate calls. IBD analysis enabled inference of pairwise identity by descent between *Plasmodium* isolates within populations. Genomic relatedness was determined by analysing the proportion, or fraction, of the genome that was IBD between *Plasmodium* isolates. Cumulative IBD across the genome was calculated using sliding window analysis (using windows of 10,000 bases) for each population. The median and range of IBD values were calculated. Thirteen of the Bijagós samples passed F_WS_ filtering (≥ 0.95) to be included in IBD analysis, and despite this small sample size, the raised IBD results appear robust.

Whole genome sequence data was interrogated for signatures of selection, using the rehh package to calculate two different statistics derived from extended haplotype homozygosity: iHS and XP-EHH^40^. These statistics are based on the decay of haplotype homozygosity, due to recombination, in the absence of selection on a particular allele^41^. Integrated haplotype score (iHS) identifies signatures of recent selection in a particular population. A negative iHS suggests that selection has favoured a derived allele, whereas a positive iHS suggests selection for the ancestral allele^41^. Cross-population extended haplotype homozygosity (XP-EHH) compares selection between two populations, through comparing the lengths of haplotypes associated with the same allele in different populations^42^. Positive XP-EHH indicates selection occurring in population A, whereas negative XP-EHH indicates selection occurring in population B. Highly variable genes (e.g., *PfEMP1*) were removed from this analysis due to the increased likelihood of them indicating false signals of selection pressure, due to an increased risk of alignment errors.

Admixture analysis was conducted using the ADMIXTURE software (version 1.3)^43^. Analysis was conducted on 387,395 SNPs with minor allele frequencies of at least 0.001. This is a program used to estimate ancestry in unrelated individuals, from autosomal SNP genotype sets. PLINK (version 1.9) was used to convert the filtered VCF file to a bed file. ADMIXTURE was then used to estimate ancestry using this bed file and a specified K value. K is the estimated number of ancestral populations. The optimum K value for ancestral admixture coefficients was estimated through cross-validation of 1–10 dimensions of eigenvalue decay and found to be K = 5. Cross-validation was repeated using 10 randomised seed inputs and results were averaged across seeds to find the optimum K value. The output was visualised in R (v3.5.1). SNPs associated with resistance to antimalarial drugs were analysed in whole genome sequence data using the dplyr package (v1.0.10) in R.

## Supplementary Information

Supplementary Information.

## Data Availability

The processed datasets are available at NCBI: http://www.ncbi.nlm.nih.gov/bioproject/905557. Bioproject: PRJNA905557. The following sample SRR accession numbers can be used to find individual sample sequence data using the SRA database search in NCBI, https://www.ncbi.nlm.nih.gov/sra/: SRR22412636, SRR22412629, SRR22412635, SRR22412622, SRR22412623, SRR22412624, SRR22412625, SRR22412626, SRR22412627, SRR22412628, SRR22412630, SRR22412631, SRR22412632, SRR22412633, and SRR22412634.
