# Supplementary material for: Population dynamics and drug resistance mutations in Plasmodium falciparum on the Bijagós Archipelago, Guinea-Bissau
Source: Sci Rep. 2023 Apr 18;13:6311. doi: 10.1038/s41598-023-33176-1 (PMC10113324; doi:10.1038/s41598-023-33176-1)
Supplement: Supplementary file 1 — Supplementary Information. [file 41598_2023_33176_MOESM1_ESM.docx]

**Supplementary Information**

Supplementary Table 1: *Study isolates (n=795) across 10 different African countries.*

| **Region** | **Country** | **Sample number (N)** |
| --- | --- | --- |
| Central Africa | Gabon | 57 |
| West Africa | Ivory Coast | 70 |
|  | The Gambia | 148 |
|  | Guinea-Bissau (Bijagós Archipelago*) | 15 |
| East Africa | Kenya | 138 |
|  | Uganda | 17 |
| Southeast Africa | Malawi | 149 |
|  | Madagascar | 24 |
| South Central Africa | Democratic Republic of the Congo | 150 |
| Horn of Africa | Eritrea | 27 |

* 15 Bijagós samples were from 5 different islands: N=7 Bubaque, N=4 Canhabaque, N=2 Formosa, N=1 Orango, N=1 Soga.

Supplementary Table 2: *F_WS_ metric assessing multiplicity of infection by assessing within-host heterozygosity (Hw) in comparison to local population heterozygosity (Hs). N = sample size.*

| **Country** | **Mean F_WS_** | **N ≥ 0.95** | **% N ≥ 0.95** |
| --- | --- | --- | --- |
| Democratic Republic of Congo | 0.820 | 56 | 37.3 |
| Eritrea | 0.951 | 22 | 78.6 |
| Gabon | 0.874 | 31 | 54.4 |
| Gambia | 0.908 | 100 | 67.6 |
| Bijagós Archipelago, Guinea-Bissau | 0.965 | 13 | 86.7 |
| Ivory Coast | 0.888 | 43 | 61.4 |
| Kenya | 0.863 | 76 | 55.1 |
| Madagascar | 0.943 | 19 | 79.2 |
| Malawi | 0.772 | 49 | 33.1 |
| Uganda | 0.788 | 7 | 41.2 |

Supplementary Table 3: *Median identity by descent (IBD) fractions and ranges for each population of P. falciparum isolates, determined using hmmIBD software.*

| **Country** | **Median IBD fraction** | **Range** |
| --- | --- | --- |
| Democratic Republic of Congo | 0.001 | 0.000 – 0.213 |
| Eritrea | 0.118 | 0.027 – 0.320 |
| Gabon | 0.005 | 0.000 – 0.244 |
| The Gambia | 0.011 | 0.004 – 0.191 |
| Guinea-Bissau (Bijagós) | 0.040 | 0.001 – 0.217 |
| Ivory Coast | 0.003 | 0.002 – 0.089 |
| Kenya | 0.012 | 0.008 – 0.161 |
| Madagascar | 0.006 | 0.000 – 0.114 |
| Malawi | 0.003 | 0.001 – 0.169 |
| Uganda | 0.067 | 0.016 – 0.187 |

Supplementary Table 4: *Integrated haplotype score* (*iHS) statistic scores for isolates from the Bijagós archipelago, N = 15. 7 SNPs* *within genes exhibited significantly high iHS scores. There were 5 positions within the SURFIN 13.1 gene; the position with the highest iHS value is displayed in the table below.*

| **Chr** | **Gene** | **Number of positions** | **Position** | **iHS value** | **Log P value** | **Ref** | **Alt** |
| --- | --- | --- | --- | --- | --- | --- | --- |
| 1 | Surface-associated interspersed protein 1.2 (SURFIN 1.2). | 1 | 513013 | -4.35 | 4.87 | A | G |
| 13 | Surface-associated interspersed protein 13.1 (SURFIN 13.1). | 5 | 106212 | 4.31 | 4.79 | G | T |

Supplementary Table 5: *Candidate regions under selection from cross-population analysis using XP-EHH scores, between isolates from the Bijagós and samples from other African regions*

| **Population comparison** | **Chromosomal region** | **Gene ID** | **Gene product** |
| --- | --- | --- | --- |
| Bijagós vs Central Africa | Chr3, 120000-150000 | PF3D7_0302500(CLAG3.1); PF3D7_0302600(ABCB4); PF3D7_0302200(CLAG3.2) | Cytoadherence linked asexual protein 3.1;  ABC transporter B family member 4, putative; Cytoadherence linked asexual protein 3.2 |
| Bijagós vs Central Africa | Chr 12, 2000000 - 2030000 | PF3D7_1248700(N/A); PF3D7_1248900(RPT6) | Conserved Plasmodium protein, unknown function;  26S protease regulatory subunit 8, putative |
| Bijagós vs Central Africa | Chr 13, 90000-120000 | PF3D7_1301700(CBP2) | CX3CL1-binding protein 2 |
| Bijagós vs East Africa | Chr 3, 120000-150000 | PF3D7_0302500(CLAG3.1); PF3D7_0302600(ABCB4); PF3D7_0302200(CLAG3.2) | Cytoadherence linked asexual protein 3.1;  ABC transporter B family member 4, putative; Cytoadherence linked asexual protein 3.2 |
| Bijagós vs East Africa | Chr 8, 410000 – 440000 | PF3D7_0808200(PMX) | Plasmepsin X |
| Bijagós vs East Africa | Chr 13, 90000 - 120000 | PF3D7_1301700(CBP2) | CX3CL1-binding protein 2 |
| Bijagós vs South Central Africa | Chr 2, 120000 – 150000 | PF3D7_0302500(CLAG3.1); PF3D7_0302600(ABCB4); PF3D7_0302200(CLAG3.2) | cytoadherence linked asexual protein 3.1;  ABC transporter B family member 4, putative; cytoadherence linked asexual protein 3.2 |
| Bijagós vs South Central Africa | Chr 11, 1170000 - 1200000 | PF3D7_1130700(SMC1); PF3D7_1130400(RPT5) | structural maintenance of chromosomes protein 1, putative;  26S protease regulatory subunit 6A, putative |
| Bijagós vs South Central Africa | Chr 12, 2000000 - 2030000 | PF3D7_1248700(N/A); PF3D7_1248900(RPT6) | conserved Plasmodium protein, unknown function;  26S protease regulatory subunit 8, putative |
| Bijagós vs South Central Africa | Chr 13, 90000 - 120000 | PF3D7_1301700(CBP2) | CX3CL1-binding protein 2 |
| Bijagós vs Southeast Africa | Chr3, 120000-150000 | PF3D7_0302500(CLAG3.1); PF3D7_0302600(ABCB4); PF3D7_0302200(CLAG3.2) | Cytoadherence linked asexual protein 3.1;  ABC transporter B family member 4, putative; Cytoadherence linked asexual protein 3.2 |
| Bijagós vs Southeast Africa | Chr 10, 1380000 - 1410000 | PF3D7_1035400(MSP3); PF3D7_1034900(MRScyt) | merozoite surface protein 3; methionine--tRNA ligase |
| Bijagós vs Southeast Africa | Chr 12, 2000000 - 2030000 | PF3D7_1248700(N/A); PF3D7_1248900(RPT6) | Conserved Plasmodium protein, unknown function;  26S rotease regulatory subunit 8, putative |
| Bijagós vs Southeast Africa | Chr 13, 90000 - 120000 | PF3D7_1301700(CBP2) | CX3CL1-binding protein 2 |
| Bijagós vs West Africa | Chr3 120000-150000 | PF3D7_0302500(CLAG3.1); PF3D7_0302600(ABCB4); PF3D7_0302200(CLAG3.2) | cytoadherence linked asexual protein 3.1; ABC transporter B family member 4, putative; cytoadherence linked asexual protein 3.2 |
| Bijagós vs West Africa | Chr 10, 1380000 - 1410000 | PF3D7_1035400(MSP3); PF3D7_1034900(MRScyt) | merozoite surface protein 3; methionine--tRNA ligase |
| Bijagós vs West Africa | Chr 12, 2000000 - 2030000 | PF3D7_1248700(N/A); PF3D7_1248900(RPT6) | conserved Plasmodium protein, unknown function;  26S protease regulatory subunit 8, putative |
| Bijagós vs West Africa | Chr 13, 90000 - 120000 | PF3D7_1301700(CBP2) | CX3CL1-binding protein 2 |

**Supplementary Table 6:** *Year and location of sample collection for publicly available genomes (malariagen.net)*

| **Country of origin** | **Site** | **Year(s) of collection** |
| --- | --- | --- |
| Democratic Rep. of Congo | Kinshasa | 2011 and 2012 |
| Eritrea | Not specified | Not specified |
| Gabon | Not specified | Not specified |
| The Gambia | Brikama and Basse | 2008 and 2014 |
| Ivory Coast | Abobo, Koumassi and Yopougon | 2013 |
| Kenya | Kilifi, Kosumu and Kombewa | 2007 and 2014 |
| Madagascar | Maevatanana, Antsohihy and Farafangana | 2012, 2013 and 2014 |
| Malawi | Chikwawa and Zomba | 2011 |
| Uganda | Apac and not specified | 2009 and 2010 |

**Supplementary Table 7:** *PfKELCH13 mutations which could not be reported on reliably due to poor sequencing coverage (*[*https://apps.who.int/iris/bitstream/handle/10665/274362/WHO-CDS-GMP-2018.18-eng.pdf*](https://apps.who.int/iris/bitstream/handle/10665/274362/WHO-CDS-GMP-2018.18-eng.pdf)*).*

| **PfKELCH13 mutations which could not be reported on reliably due to poor sequencing coverage of the PfKELCH13 gene.** |
| --- |
| Validated: F446I, M476I, Y493H, R539T, I543T, P553L, R561H, C580Y, C469Y |
| Candidate/associated: P441L, G449A, C469F, A481V, P527H, N537I/D, G538V, V568G, P574L, F673I, A675V |

*
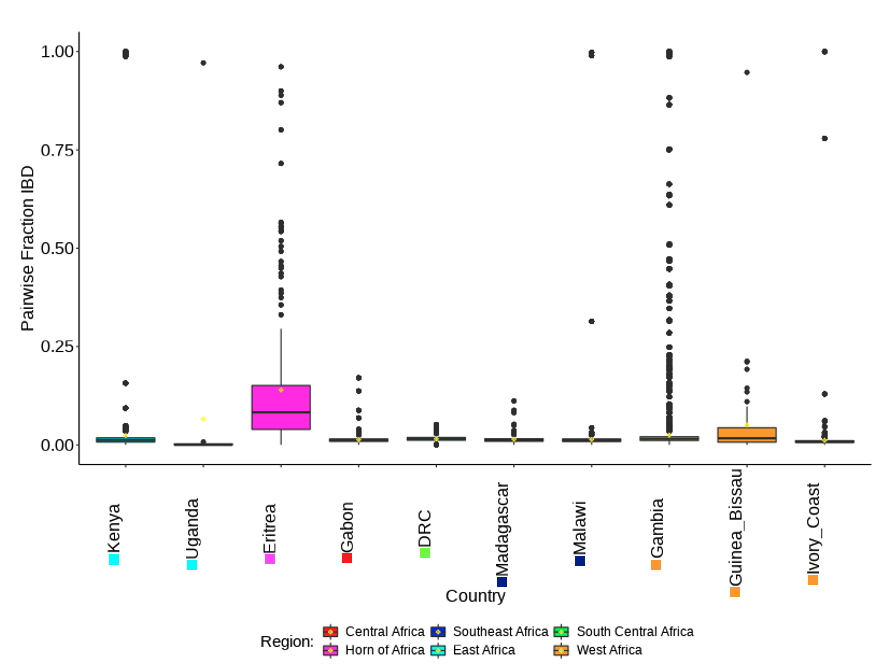
*

Supplementary Figure 1: *A boxplot showing the pairwise identity by identity by descent (IBD) fraction for P. falciparum isolates from each of the countries analysed.*


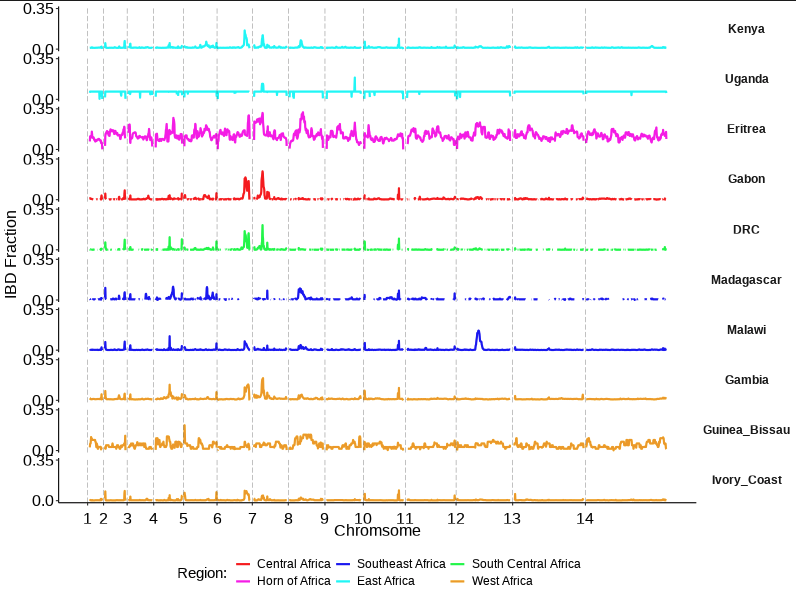


Supplementary Figure 2: *Identity by descent (IBD) fractions along the genome for P. falciparum isolates from each of the countries analysed.*


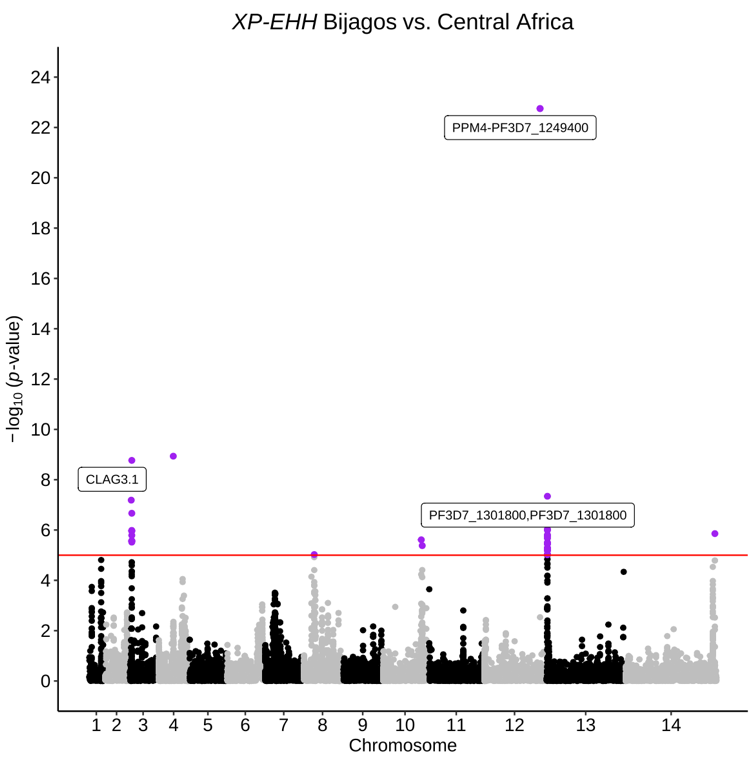

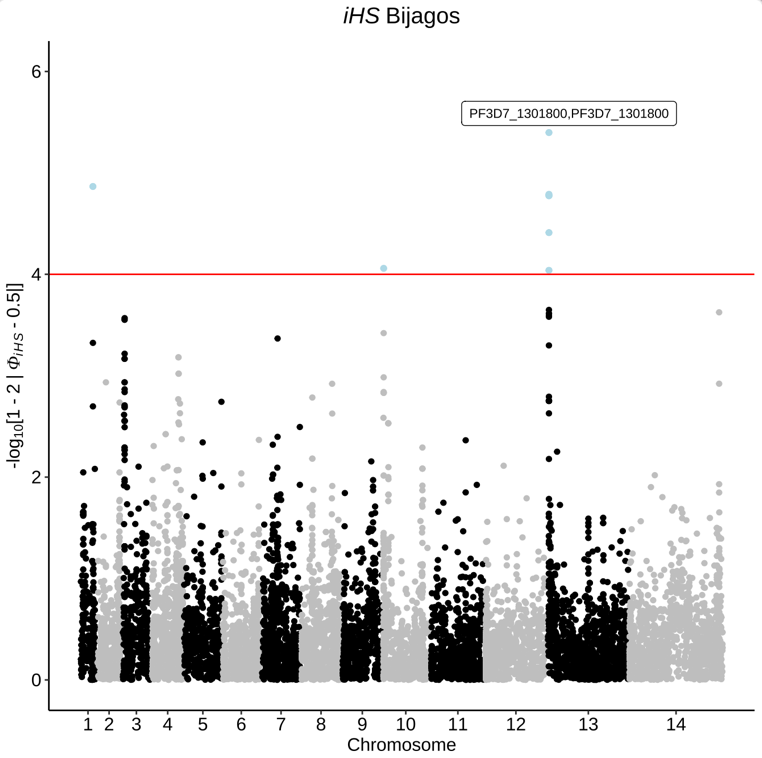


**A**

**B**

Supplementary Figure 3: *Manhattan plots showing A) iHS scores for isolates from the Bijagós, Guinea-Bissau, B) XP-EHH scores from cross-population analysis between isolates from the Bijagós and isolates from West Africa, from The Gambia and Ivory Coast*

**
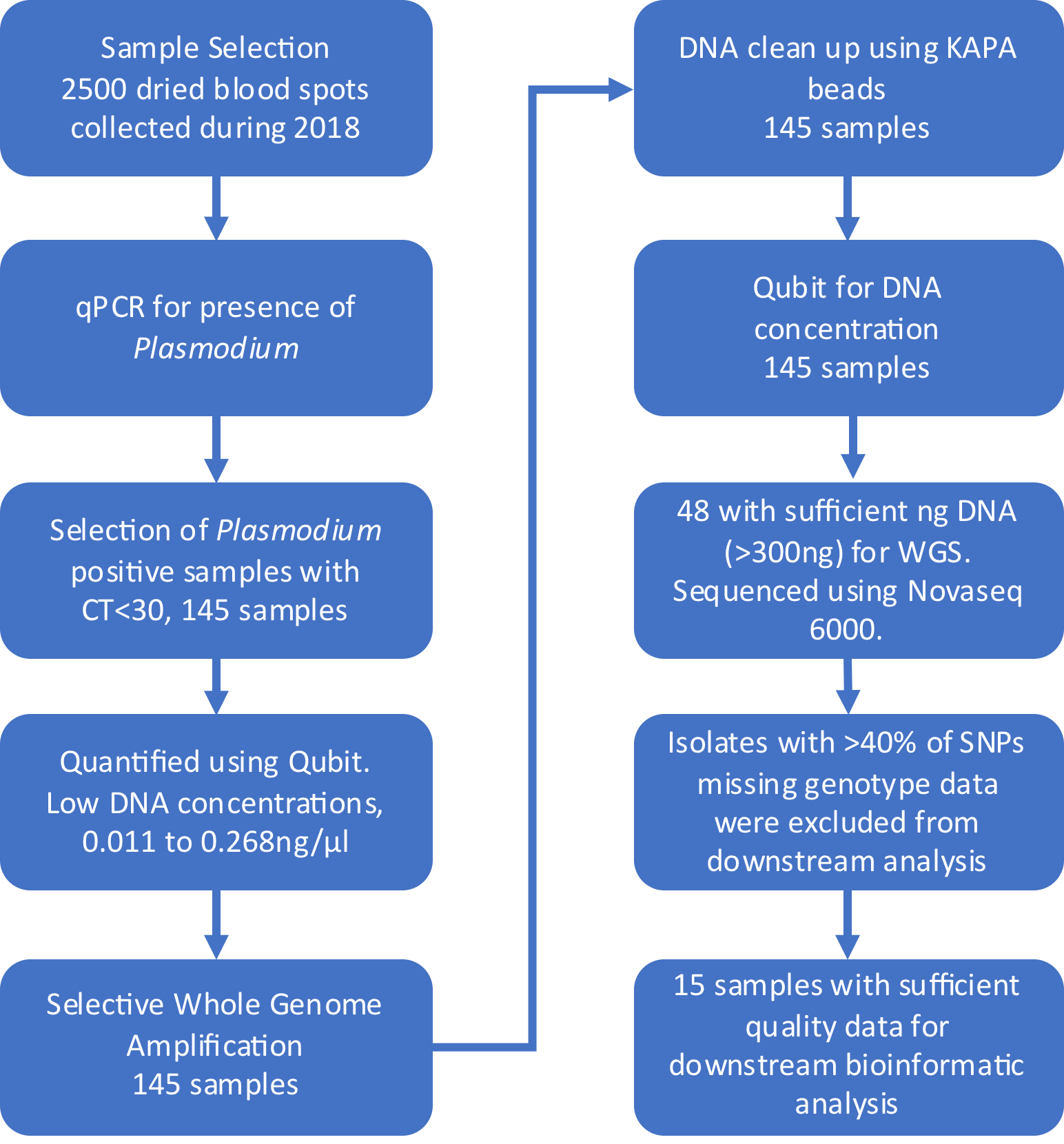
**

**Supplementary Figure 4:** *Filtering steps for P. falciparum isolates for inclusion in genomic analyses.*
